# Non-Destructive Early Sex Identification of Embryonated Quail Eggs Using Raman Spectroscopy

**DOI:** 10.3390/ani16111737

**Published:** 2026-06-05

**Authors:** Qian Yan, Zesheng Wang, Zhoushi Tan, Jiaquan Wu, Qiaohua Wang

**Affiliations:** 1 College of Engineering, Huazhong Agricultural University, Wuhan 430070, China; yqyq@webmail.hzau.edu.cn (Q.Y.); wangzesheng@webmail.hzau.edu.cn (Z.W.); 2068409343@webmail.hzau.edu.cn (Z.T.); ggbond@webmail.hazu.edu.cn (J.W.); 2 Ministry of Agriculture Key Laboratory of Agricultural Equipment in the Middle and Lower Reaches of the Yangtze River, Wuhan 430070, China; 3 National Research and Development Center for Egg Processing, Huazhong Agricultural University, Wuhan 430070, China

**Keywords:** embryonated quail eggs, GA-ELM model, non-destructive testing, Raman spectroscopy, sex identification

## Abstract

Routine culling of day-old male chicks in the global poultry industry triggers severe resource waste and critical animal welfare crises, while non-destructive early sex identification techniques for embryonated quail eggs remain a prominent research gap. This study developed a novel Raman spectroscopy-based method for quail embryo sexing on incubation day 5, using an innovative “shell perforation without inner membrane damage” sampling strategy. The optimized GA-CARS-ELM model achieved 80.95% accuracy on the independent test set with 0.39 ms single-sample model inference time and 5.3 ± 0.5 min total per-egg processing time under manual operation, outperforming mainstream machine learning and deep learning algorithms. This work fills the relevant research gap and provides vital technical support for the development of automated pre-hatching sex sorting systems in poultry farming.

## Simple Summary

Every year, billions of newly hatched male chicks are culled worldwide in the poultry industry because they cannot lay eggs, causing severe waste of resources and serious animal welfare concerns. However, there is still no effective way to identify the sex of quail embryos early before hatching. This study developed a new method to determine the sex of quail embryos on the 5th day of incubation without harming them. We made a tiny hole in the eggshell without damaging the inner membrane, then analyzed the molecular signals from the inner membrane to distinguish male and female embryos. The method achieved an accuracy of over 80%. This technology allows farmers to sort eggs before hatching, avoiding the culling of male chicks. It will help reduce resource waste, improve animal welfare, and promote more sustainable quail farming.

## 1. Introduction

The poultry industry is a core pillar of the agricultural and rural economy worldwide, with continuously expanding market demand for poultry products [1]. As a characteristic small-scale poultry species, quail has become an indispensable component of the global poultry farming system, owing to its short incubation cycle, high reproductive efficiency, and low breeding cost [2]. In egg-type poultry production systems, female individuals serve as the core production units, while male chicks have no economic value and are routinely culled immediately after hatching [3]. Globally, more than 7 billion day-old male chicks are culled annually to meet egg production demands, a practice that not only causes severe waste of hatching resources, feed raw materials, and labor, but also triggers widespread animal welfare and ethical controversies, which has become a critical bottleneck restricting the high-quality development of the global poultry industry [4]. Since Germany, France, and multiple other countries have legislated to ban the systematic culling of day-old male chicks, early, non-destructive, and high-throughput in ovo sex identification technology has become an urgent industrial demand worldwide.

Traditional poultry sex identification methods primarily rely on post-hatching techniques, including anal vent sexing [5], feather color sexing [6], and feather growth rate sexing [7]. However, these methods have prominent limitations in practical application: anal vent sexing has stringent technical requirements for operators, involves intensive labor, and easily causes stress injury to chicks; feather color and feather growth rate-based methods are only applicable to specific specialized breeds with matching genetic traits, severely restricting their widespread application. To address these limitations, numerous in ovo sex identification technologies during incubation have been explored in recent decades. Molecular biological methods, such as PCR [8] and hormone detection [9], achieve high detection accuracy, yet they require invasive sampling, complex operation procedures, and high testing costs. These drawbacks frequently lead to embryo mortality and reduced hatchability, making them incompatible with high-throughput online detection in large-scale commercial farming. Optical detection technologies, including near-infrared spectroscopy [10,11] and hyperspectral imaging [12,13,14,15], have been widely investigated for non-destructive testing, but they suffer from severe signal interference from eggshell matrices, difficulty in extracting weak sex-related features, and insufficient model generalizability across breeds and batches, failing to meet the accuracy requirements for industrial production.

Raman spectroscopy [16], as a non-destructive detection technique at the molecular level, exhibits unique advantages including minimal water interference, high molecular specificity, and rapid detection speed, and has shown promising application potential in biological information detection of avian embryonated eggs. Nevertheless, existing studies on Raman spectroscopy-based in ovo sexing have almost exclusively focused on chicken embryos [17,18,19], with a striking paucity of research on early non-destructive sex identification of embryonated quail eggs. More critically, most relevant studies prioritize the optimization of model classification performance, with insufficient in-depth elucidation of the biological mechanisms underlying the sex-specific spectral discrimination signals, which limits the fundamental understanding and further iterative optimization of the detection technology.

To fill the above research gaps, this study took embryonated Korean quail eggs as the research object, and selected day 5 of incubation as the detection window. A novel “shell perforation without inner membrane damage” sampling strategy at the blunt end of the egg was adopted to collect Raman spectral data of the inner shell membrane. We optimized the full workflow of spectral data processing including data augmentation, preprocessing, and characteristic wavelength selection, constructed a sex discrimination model using an extreme learning machine (ELM) optimized by genetic algorithm (GA), and elucidated the potential biological connotation of sex-related characteristic signals through Raman peak assignment. The performance of the proposed model was further compared with mainstream machine learning and deep learning models. This study aims to establish a non-destructive early sex identification method for quail embryos with high accuracy, rapid detection speed, and strong generalizability, to provide technical support for pre-hatching sex sorting in the poultry farming industry, and to offer a reference for non-destructive detection of other avian embryonated eggs.

## 2. Materials and Methods

### 2.1. Experimental Materials

The embryonated Korean quail eggs used in this study were sourced from a standardized large-scale quail breeding base in Jiangxi Province, China. All eggs were laid by 35–40-week-old healthy breeding quail with consistent genetic background, stable fertilization rate and reliable hatching performance. A total of 550 qualified fresh quail eggs were procured; after pre-incubation visual inspection and weight grading, 42 unqualified samples (broken, malformed, or with uneven eggshell thickness) were excluded. Finally, 508 eggs were formally incubated for subsequent spectral acquisition and model training, validation and testing.

### 2.2. Instruments and Reagents

The main instruments and reagents used in this study were as follows: Thermo Scientific™ DXR3 micro-Raman spectrometer (Thermo Fisher Scientific, Waltham, MA, USA); intelligent fully automatic incubator (Weiya Incubation Equipment Manufacturing Co., Ltd., Dezhou, China); ecological brooding incubator (Jiahe Incubation Equipment Manufacturing Co., Ltd., Dezhou, China); micro precision punch (Shanghai Precision Instrument Factory, Shanghai, China); SW-CJ-1F laminar flow ultra-clean workbench (Shangdao Instrument Manufacturing Co., Ltd., Shanghai, China); and sterile air-permeable sealing tape, disposable sterile wipes, 5% benzalkonium bromide disinfectant, and anhydrous ethanol.

### 2.3. Experimental Design and Spectral Acquisition

#### 2.3.1. Determination of Detection Window and Sampling Position

Combined with the gonadal differentiation pattern of quail embryos, expression characteristics of key sex differentiation-related genes and pre-experiment results, day 5 of incubation was determined as the spectral acquisition time point. Molecular biological studies have confirmed that days 4–5 of incubation are the critical window for quail embryonic sex differentiation, during which key sex differentiation genes including *P450arom*, *ER* and *3β-HSD* exhibit extremely significant sex-biased expression differences between male and female embryos [20], forming stable and detectable sex-specific molecular signals to support Raman spectral feature capture.

**Figure 1:**
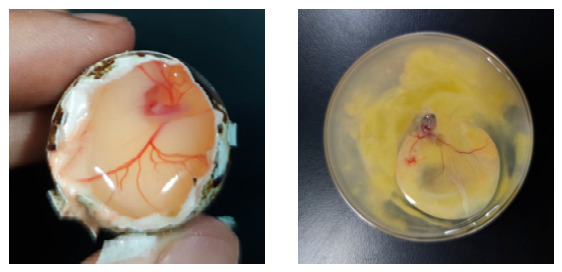
Quail embryos on day 5 of incubation.

The avian embryonic nervous system is initially formed on day 7 of incubation [3], so testing on day 5 causes minimal interference to embryonic development, maximizes embryo survival rate and complies with animal welfare principles; this window also ensures good light transmittance at the egg blunt end, avoiding blood vessel-induced spectral interference. The blunt end (air chamber end) was determined as the sampling position, for its uniform eggshell thickness minimizes mechanical damage to embryos, and the inner shell membrane here can stably enrich sex differentiation-related metabolites for stable and repeatable signal acquisition. Quail embryos on day 5 of incubation are shown in Figure 1.

#### 2.3.2. Sample Pretreatment

**Figure 2:**
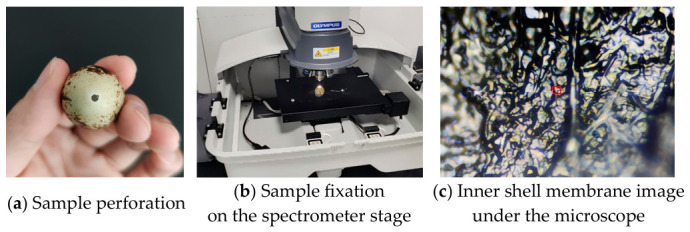
Sample preparation and spectral acquisition: (**a**) Sample perforation at the blunt end of the egg; (**b**) vertical fixation of the perforated egg on the spectrometer stage; (**c**) microscopic image of the intact inner shell membrane for spectral measurement.

On day 5 of incubation, eggs were removed from the incubator, and all operations were performed under aseptic conditions in a laminar flow ultra-clean workbench throughout the process. A micro precision punch was used to grind a circular small hole with a diameter of approximately 3 mm at the center of the blunt end of the egg (Figure 2a), with the grinding depth strictly controlled to achieve “shell perforation without inner membrane damage” (only the calcium carbonate layer of the eggshell was completely removed, while the underlying inner shell membrane remained fully intact). This strategy differs fundamentally from existing Raman-based in ovo sexing methods for chicken embryos [17,18,19], which typically penetrate the inner shell membrane to directly irradiate embryonic tissues or allantoic fluid, leading to higher embryo mortality. After perforation, eggshell debris was cleaned with sterile wipes, the opening was immediately sealed with sterile air-permeable tape, and the eggs were returned to the incubator and left to stand for 2 h. Raman spectral acquisition was performed after the internal environment of the embryonated egg was stabilized and the embryo resumed normal development. The average time required for manual sample pretreatment (perforation, cleaning and sealing) was 1.1 ± 0.3 min per egg.

#### 2.3.3. Spectral Acquisition

All spectral acquisition was performed in a 25 °C constant-temperature light-proof dark room to eliminate interference from ambient light, temperature fluctuations and mechanical vibration. Prior to acquisition, the Raman spectrometer was calibrated with a silicon standard to ensure wavenumber deviation < 0.5 cm^−1^. During acquisition, the perforated end of eggs was fixed vertically upward on the spectrometer stage (Figure 2b), and the 785 nm laser was focused on the inner shell membrane via a 50× objective lens (Figure 2c) to avoid vascular signal interference. Core acquisition parameters are detailed in Table 1.

Three parallel spectra were collected per sample, with the average taken as the original spectral data. Post-acquisition, the egg opening was re-sealed with sterile tape, and numbered eggs were returned to the incubator, with their hatching and survival status fully tracked throughout incubation. The total per-egg processing time including pretreatment, 2 h stabilization, spectral acquisition and postprocessing was 5.3 ± 0.5 min under manual operation.

### 2.4. Validation of True Sex Labels

**Figure 3:**
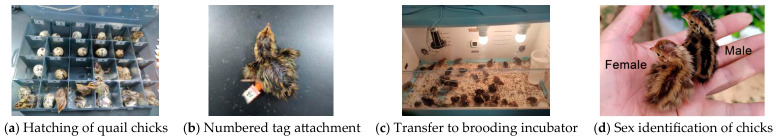
Acquisition of true sex labels: (**a**) Normal hatching of quail chicks on day 17; (**b**) attachment of numbered tags to maintain sample traceability; (**c**) transfer of hatched chicks to the ecological brooding incubator; (**d**) feather color-based sex identification within 24 h after hatching.

On day 16 of incubation, eggs were transferred to hatching trays with individually numbered compartments to ensure one-to-one correspondence between hatched chicks and spectral sample numbers and avoid sample confusion. Sex identification was performed within 24 h after hatching using the feather color sexing method: Korean quail has clear sex-linked genetic characteristics, where male chicks have dark black back feathers, and female chicks have light yellow back feathers, enabling rapid and accurate validation [21] (Figure 3). The final sex labeling rule was as follows: male was labeled as 1, and female was labeled as 0.

During the experiment, samples with preparation damage, unfertilized eggs, mid-term dead embryos, and abnormal spectral data (signal loss, severe baseline drift, abnormal peak value) were excluded, and the remaining valid samples were used for subsequent spectral data processing and model training, validation and testing.

### 2.5. Spectral Data Processing and Modeling Methods

#### 2.5.1. Spectral Data Augmentation

To mitigate model overfitting and insufficient generalizability under small sample conditions, three data augmentation schemes were established to expand only the training set samples: left–right shift method, Gaussian noise superposition method, and a method combining both.

First, valid original samples were divided into training set, validation set, and test set at a ratio of 6:2:2 using the Kennard-Stone algorithm [22], ensuring a balanced sex ratio and consistent original spectral feature space distribution among the three datasets. The validation and test sets retained original spectral data throughout, without participating in any augmentation operations, and were only used for model parameter optimization and unbiased generalizability evaluation.

Subsequently, data augmentation was performed exclusively on the training set and prior to any spectral preprocessing. The left–right shift method was implemented by randomly shifting each original spectrum within a range of ±5 cm^−1^ along the wavenumber axis, with missing values filled using linear interpolation. The Gaussian noise superposition method added zero-mean Gaussian noise with a standard deviation of 0.01 times the maximum spectral intensity to each spectrum. Each original training sample was augmented 5 times using the optimal scheme, resulting in a 5-fold expansion of the training set size. The structural similarity (SSIM) was used to evaluate feature consistency between augmented and original spectra, and the classification accuracy of the ELM model on the validation set was taken as the core indicator to screen the optimal data augmentation scheme.

#### 2.5.2. Spectral Preprocessing and Characteristic Wavelength Selection

**Figure 4:**
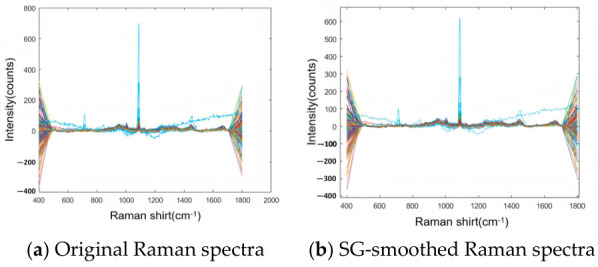
Waveform comparison of original and preprocessed Raman spectra: (**a**) Original Raman spectra of samples; (**b**) Raman spectra after SG smoothing preprocessing.

Original Raman spectra are susceptible to interference from baseline drift, random high-frequency noise and light scattering effects (Figure 4). To highlight sex-related molecular feature information, this study compared the correction effects of three mainstream spectral preprocessing methods: Savitzky–Golay (SG) smoothing, standard normal variate (SNV) transformation, and multiplicative scatter correction (MSC) [23]. SG smoothing was set with a window width of 11 and polynomial order of 3 to suppress noise while retaining characteristic peaks; SNV and MSC were performed following standard workflows to correct scattering interference from sample inhomogeneity and optical path differences.

**Figure 5:**
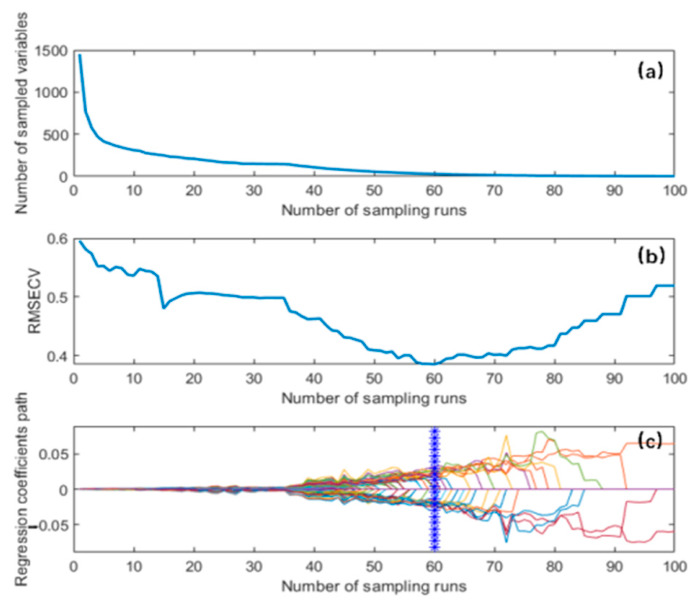
Screening of characteristic wavelengths via the CARS algorithm: (**a**) Number of retained variables with increasing sampling runs; (**b**) RMSECV values across different sampling runs; (**c**) regression coefficient paths of all wavelength variables.

**Figure 6:**
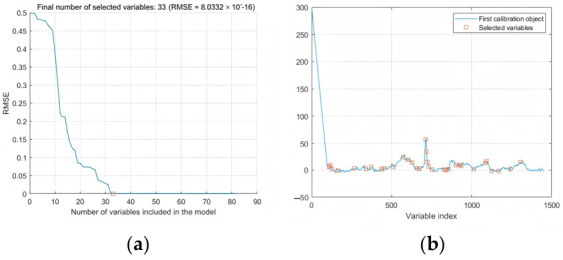
Screening of characteristic wavelengths via the SPA: (**a**) RMSE values with increasing number of selected variables; (**b**) distribution of the 33 selected characteristic wavelengths in the full spectral range.

The Raman spectrum of quail embryonated eggs contains 1453 wavelength variables in the 400–1800 cm^−1^ range, most of which are irrelevant to sex information, and direct modeling is prone to the “curse of dimensionality”. Two methods, competitive adaptive reweighted sampling (CARS) [24] and successive projections algorithm (SPA) [25], were used to extract sex-related characteristic wavelengths for dimensionality reduction. Importantly, both CARS and SPA were applied exclusively to the training set to avoid data leakage. The characteristic wavelength indices selected from the training set were then used to extract corresponding features from the validation and test sets, ensuring methodological consistency and unbiased model evaluation. If applied to the entire dataset, the feature selection process would incorporate information from the test set, leading to overestimated model performance. The CARS algorithm was set with 100 Monte Carlo sampling runs and 5-fold cross-validation, and finally screened 29 characteristic wavelengths at the 60^th^ sampling run with the minimum root mean square error of cross-validation (RMSECV) (Figure 5); the SPA screened 33 characteristic wavelengths with the minimum RMSECV as the termination condition (Figure 6). The final input vector for classification consisted of the spectral intensity values at the selected characteristic wavelengths (or full wavelengths for baseline models).

#### 2.5.3. Construction of Extreme Learning Machine Model

In this study, an extreme learning machine (ELM) [26] was employed to construct a binary classification model for sex discrimination of quail embryos. As a single-hidden-layer feedforward neural network, the ELM does not require iterative adjustment of weights and thresholds between the input layer and the hidden layer. It can derive the optimal analytical solution of the output layer weights solely by solving the generalized inverse matrix, which is suitable for rapid classification and recognition of high-dimensional Raman spectral data.

The constructed ELM model adopted a three-layer feedforward network structure consisting of an input layer, a single hidden layer, and an output layer. The number of neurons in the input layer was fully matched with the dimension of input variables, which were divided into three categories: full-wavelength spectral data, and characteristic wavelength data screened by the competitive adaptive reweighted sampling (CARS) and successive projections algorithm (SPA) algorithms. For the hidden layer, the weight matrix *W* from the input layer to the hidden layer and the neuron threshold *b* were randomly generated during model initialization and remained fixed throughout the training process. Nonlinear mapping of the input data was completed via the activation function *g*(∙), with the calculation formula of the hidden layer output matrix shown in Formula (1).

(1) $$H=g\left(W\cdot X+b\right)$$

The output layer was configured with 1 neuron for the binary sex classification task of quail embryos, which output the classification results of male (labeled as 1) and female (labeled as 0). The weight matrix *β* from the hidden layer to the output layer was solved by the least square method without iterative optimization, with the calculation formula shown in Formula (2).

(2) $$\hat{\beta }=H^{+}\cdot T$$

where *H*^+^ is the Moore–Penrose generalized inverse of *H*, *T* is the sample sex label matrix, and $\hat{\beta }$ is the optimal analytical solution of the output layer weights.

With the classification accuracy of the validation set as the core evaluation indicator, we first compared the classification effects of the Sigmoid and Hardlim activation functions. Subsequently, we traversed the number of hidden layer neurons in the range of 20–1500 with a step size of 20. Each parameter group was independently and repeatedly trained 10 times, and the average accuracy of the validation set was used to eliminate random fluctuations and determine the optimal hyperparameter combination of each model.

#### 2.5.4. Model Optimization Based on Genetic Algorithm

**Figure 7:**
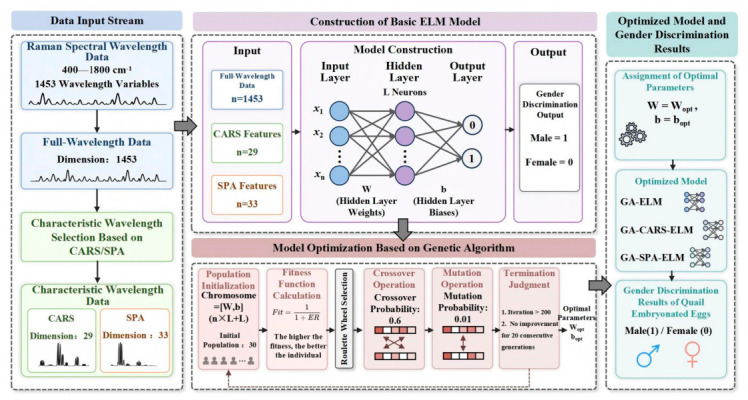
Structure diagram of the GA-optimized ELM model for sex discrimination of quail embryos.

The input layer weights and hidden layer thresholds of the pre-constructed ELM models were randomly generated during initialization. While this design guaranteed high training efficiency, it also resulted in strong parameter randomness, insufficient model stability, and fluctuating generalization performance in practical application scenarios. To address these limitations, we performed global optimization on the core parameters of the ELM models using a genetic algorithm (GA) [27] in this study. GA is a stochastic global search optimization algorithm that simulates biological evolution and natural selection; it achieves the survival of the fittest via selection, crossover, and mutation operations, and can rapidly locate the global optimal solution in complex solution spaces while effectively avoiding local optimum issues. The core optimization targets of the GA were the input layer weight matrix W and hidden layer neuron threshold b of the ELM model. With the minimization of the model’s classification error rate on the validation set as the optimization objective, we identified the optimal parameter combination through iterative population evolution, and constructed three optimized models: GA-ELM, GA-CARS-ELM, and GA-SPA-ELM. The structural framework of the GA-optimized ELM model is shown in Figure 7.

The overall optimization workflow of the GA-ELM model was divided into 6 core steps, with detailed parameter settings as follows:

(1) Parameter coding and population initialization: Real number coding was adopted, and the input weight *W* and hidden layer threshold *b* to be optimized were spliced into a single chromosome (individual). The chromosome length was determined by the number of input layer neurons n and hidden layer neurons *L*, with a total length of *n* × *L* + *L*. The number of individuals in the initial population was set to 30, and the initial population was randomly generated within the parameter value range to complete optimization initialization.
(2) Fitness function calculation: The fitness function was constructed with the ELM model’s classification error rate on the validation set as the core, with the fitness value negatively correlated with the classification error rate (calculation formula shown in Formula (3)).
  (3) $$Fitness=\frac{1}{1+ErrorRate}$$
  where ErrorRate is the sex classification error rate of the ELM model on the validation set. The parameters of each individual in the population were assigned to the ELM model, and the fitness value of the corresponding individual was calculated after model training to complete the fitness evaluation of the population.
(3) Selection, crossover, mutation operations and termination judgment: The roulette wheel selection method was adopted for the selection operation. The crossover probability was set to 0.6, with a real number crossover method used for parent individuals to exchange gene fragments and generate offspring. The mutation probability was set to 0.01, with random mutation performed on selected chromosome gene sites within the parameter range to avoid local optimum. The maximum genetic generation was set to 200; iteration was terminated when the maximum generation was reached, or the optimal population fitness showed no significant improvement for 20 consecutive generations. The individual with the highest fitness value was output, and the decoded optimal parameters were assigned to the ELM model to complete the final construction of the optimized models.

#### 2.5.5. Model Evaluation Metrics

We adopted five core quantitative indicators to comprehensively evaluate the discrimination performance, generalization ability, and industrial applicability of all constructed models. The primary indicators included classification accuracy, precision, recall, F1-score, and single-sample discrimination time. The calculation formulas are shown in Formula (4).

(4) $$Accuracy=\frac{TP+TN}{TP+TN+FP+FN}\times 100\%$$

where *TP* and *TN* represented correctly classified female and male samples, while *FP* and *FN* represented misclassified male and female samples, respectively. We used the classification accuracy on the independent test set to assess model generalizability to unknown samples, and recorded the single-sample discrimination time via the MATLAB R2023a built-in timing function to evaluate the model’s adaptability to industrial online detection.

## 3. Results and Analysis

### 3.1. Statistics of Hatching Status and Valid Samples

**Figure 8:**
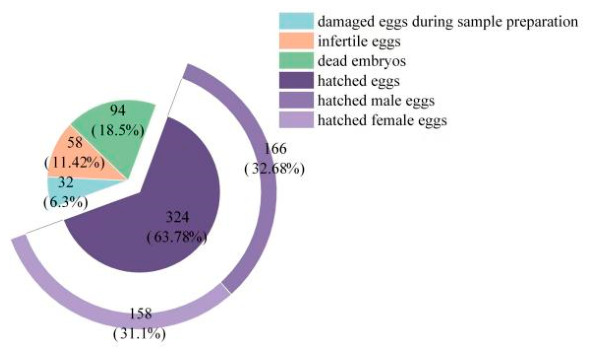
Statistics of sample hatching status.

The preparation damage, unfertilized eggs, dead embryos, and hatching status of the 508 incubated eggs were tracked and recorded one by one throughout the experiment, and the results are shown in Figure 8.

A total of 324 chicks hatched normally in the experiment, including 166 males and 158 females. The number of male and female samples was basically equal, which conformed to the natural sex ratio of 1:1 in avian incubation, with no significant sex bias, ensuring the sample balance for subsequent model training. The final hatching rate of this experiment was 63.78%, which was lower than the normal hatching level of Korean quail. The main reasons were as follows: to achieve effective spectral acquisition, drilling preparation was required at the blunt end of the embryonated egg, which easily caused damage to the inner shell membrane and egg liquid leakage during the grinding process, directly leading to the failure of 32 samples during preparation. Meanwhile, repeated tearing of the sealing tape during spectral acquisition and ambient temperature fluctuations between the ultra-clean workbench and the incubator also aggravated embryonic stress and increased the embryonic mortality rate. Notably, the hatching rate achieved with our “shell perforation without inner membrane damage” strategy was significantly higher than that of existing membrane-penetrating Raman methods (reported by Galli et al. [17]), with a statistically significant difference (*p* < 0.05, chi-square test).

After excluding samples with preparation damage, unfertilized eggs, dead embryos, and 11 samples with abnormal spectral data (signal loss, severe baseline drift, abnormal peak value), a total of 313 valid modeling samples were finally obtained, including 158 male samples and 155 female samples. The distribution of valid samples across the three datasets is shown in Table 2.

### 3.2. Optimization Results and Analysis of Key Steps in Spectral Data Processing

**Figure 9:**
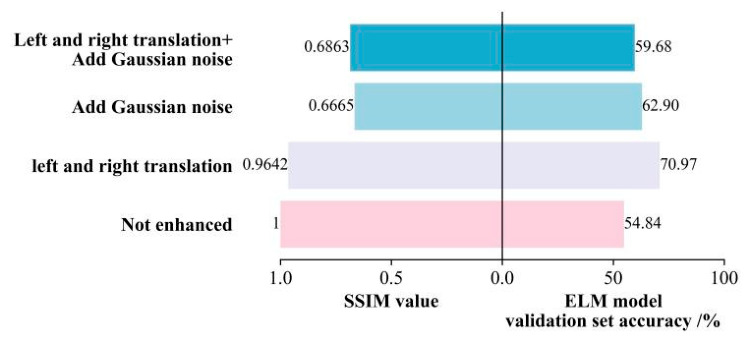
Performance comparison of different data augmentation methods.

To address the overfitting issue of model construction under small sample conditions, we compared the performance of three data augmentation methods, with the results shown in Figure 9. When no augmentation was applied to the training set, the validation set accuracy of the ELM model was only 54.84%, indicating that the model had almost no effective discrimination ability, and its generalization performance was severely limited due to insufficient valid samples for learning sex-related spectral features. The results showed that the left–right shift method was the optimal data augmentation scheme, with the highest spectral structural similarity (SSIM) value of 0.9642. This method maximally retained the core information of the original spectrum while expanding the training set samples, and increased the validation set accuracy of the model by 16.13 percentage points compared with the non-augmented group, which was significantly better than the Gaussian noise superposition augmentation methods. For the two schemes with Gaussian noise superposition, the additionally introduced noise masked some weak sex-related feature signals due to the weak original Raman signal of quail embryonated eggs, resulting in a significant decrease in spectral similarity and model discrimination accuracy. Finally, the left–right shift combined with linear interpolation filling method was adopted to expand the valid samples of the training set by five times, which effectively alleviated the overfitting problem of small sample modeling.

Biologically, the left–right shift augmentation simulates the small wavenumber drifts that commonly occur in Raman spectrometers during long-term operation and the slight positional variations in samples on the measurement stage [28,29]. These variations are inevitable in industrial production environments, and training the model with augmented spectra enhances its robustness to real-world measurement noise and instrumental fluctuations by accounting for these common instrumental artifacts.

To eliminate spectral interference and highlight sex-related molecular features, we compared the improvement effects of three mainstream preprocessing methods on model performance, with the results shown in Table 3. After SG smoothing preprocessing, the validation set accuracy of the ELM model increased from 70.97% to 72.58%, which was the only scheme that achieved accuracy improvement among the three preprocessing methods (*p* = 0.032, paired *t*-test). In contrast, SNV and MSC led to the loss of weak sex-related feature information due to overcorrection, and eventually reduced the discrimination accuracy of the model. Therefore, SG smoothing (window width 11, polynomial order 3) was determined as the optimal spectral preprocessing method for this experiment.

To address the high redundancy of 1453 wavelength variables in the full spectrum, we used CARS and SPA to extract sex-related characteristic wavelengths. The results showed that both feature selection algorithms could effectively eliminate redundant irrelevant variables in the spectrum, and increased the validation set accuracy of the model by 6.45 and 4.83 percentage points, respectively (*p* < 0.05 for both). Based on the comprehensive variable compression effect and model discrimination accuracy, the 29 characteristic wavelengths screened by the CARS algorithm were preferentially used for subsequent model construction and optimization.

### 3.3. Processing Parameter Optimization of ELM Model and Performance Analysis of GA Optimization

The discrimination performance of the ELM model is mainly governed by two core parameters: the hidden layer activation function and the number of hidden layer neurons. With the classification accuracy of the validation set as the core evaluation indicator, we traversed the number of neurons in the range of 20–1500 with a step size of 20, and compared the fitting effects of two mainstream activation functions, Sigmoid and Hardlim. Finally, we determined the optimal parameter combinations for the three types of models: full-wavelength ELM, CARS-ELM, and SPA-ELM.

Parameter optimization results showed that the Sigmoid activation function exhibited superior fitting performance across all three model types, as its nonlinear mapping capability was more suitable for the feature learning requirements of the binary sex classification task for quail embryos, and could more accurately capture the nonlinear correlation between spectral data and embryo sex. The optimal number of hidden layer neurons for the models after CARS and SPA feature selection was only 40, which was much lower than 800 for the full-wavelength ELM model. The core reason was that feature selection eliminated redundant irrelevant variables in the spectrum, greatly reduced the input dimension, and completed core feature fitting without a large number of neurons, which structurally reduced the risk of model overfitting.

**Figure 10:**
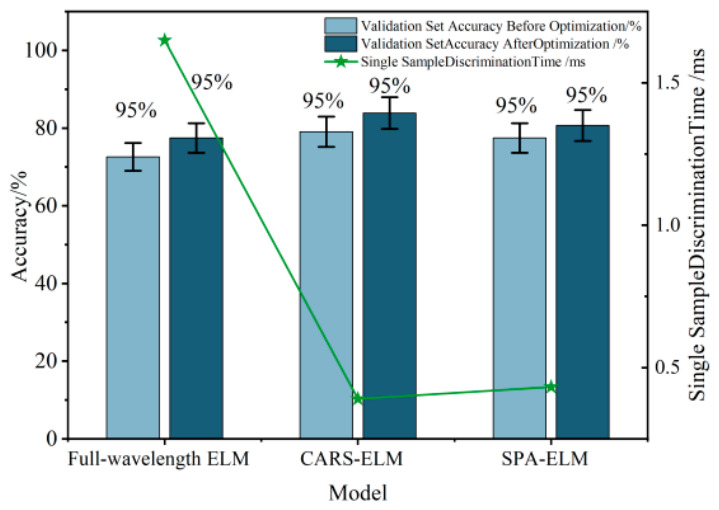
Performance comparison of models before and after GA optimization (mean ± 95% CI, *n* = 10).

Although the basic ELM model constructed with the above optimal parameters had basic discrimination capability, the random generation of input weights and thresholds led to random fluctuations in model performance and insufficient generalization ability. We therefore performed global optimization on the input layer weights and hidden layer thresholds of the three ELM models using the GA, with the comparison of validation set accuracy and single-sample discrimination time before and after optimization shown in Figure 10. All results are presented as mean ± 95% confidence interval (CI) based on 10 independent repeated runs.

The results showed that after GA optimization, the validation set accuracy of all three ELM models was significantly improved, with effectively enhanced model stability and generalization ability. Among them, the GA-CARS-ELM model achieved the best comprehensive performance, with a validation set accuracy of 83.87% ± 1.61% (4.84 percentage points higher than that before optimization) and a single-sample discrimination time of only 0.390 ms, less than 1/4 of that of the full-wavelength GA-ELM model.

**Figure 11:**
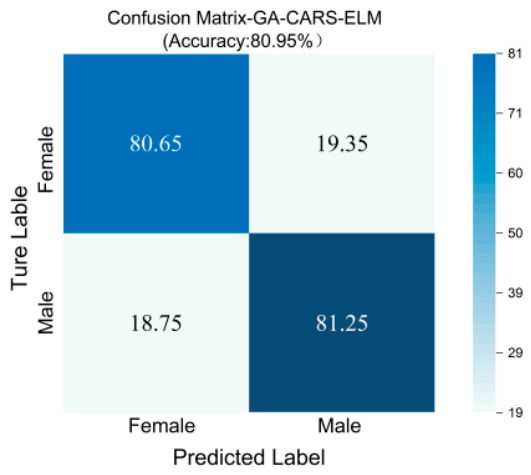
Confusion matrix of the GA-CARS-ELM model on the test set (mean ± 95% CI, *n* = 10).

Further confusion matrix analysis was performed on the classification results of the GA-CARS-ELM model on the test set (Figure 11). The model achieved 81.25% ± 3.13% discrimination accuracy for male samples and 80.65% ± 3.23% for female samples on the test set, with highly balanced accuracy between the two sexes and no significant category bias. This indicated that the GA-optimized model had a stable and efficient recognition ability for sex-related feature signals of both male and female quail embryos, and eliminated the fluctuation of classification results caused by parameter randomization of the basic ELM model. The comprehensive performance metrics of the GA-CARS-ELM model on the test set were: accuracy 80.95% ± 1.59%, precision 80.65% ± 3.23%, recall 80.65% ± 5.65%, and F1-score 80.65% ± 2.93% (mean ± 95% confidence interval, *n* = 10 independent runs).

### 3.4. Comparative Analysis of Performance of Different Machine Learning Models

To comprehensively verify the comprehensive performance advantages of the constructed GA-CARS-ELM model in the non-destructive sex identification task of quail embryos, we selected four classic traditional machine learning models [30] and three mainstream deep learning models [31] for horizontal comparison, covering mainstream algorithm architectures in the spectral classification field including linear classification, ensemble learning, fully connected neural networks, and convolutional networks.

**Figure 12:**
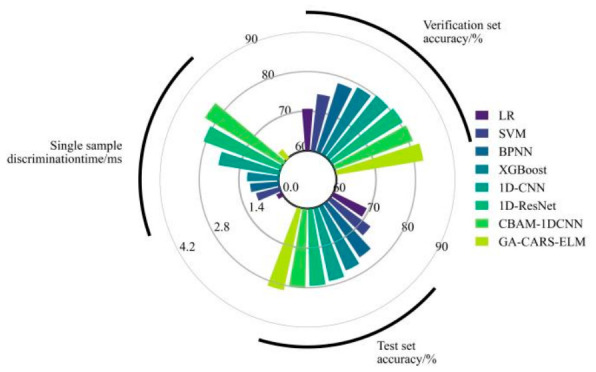
Performance comparison of different machine learning and deep learning models (CARS input dataset).

All comparison models adopted exactly the same dataset input (29-dimensional characteristic wavelengths after SG smoothing preprocessing and CARS feature selection), the same 6:2:2 training set/validation set/test set division rules, and an identical training environment. We used independent test set accuracy and single-sample discrimination time as core evaluation indicators, with the detailed numerical results shown in Table 4 and the radar chart comparison shown in Figure 12.

For traditional machine learning models, the linear Logistic Regression (LR) model had an extremely fast inference speed, but its discrimination accuracy was at the lowest level among all models due to the limitation of its algorithm architecture, which could not fully fit the nonlinear correlation between spectral data and embryo sex. The classic Support Vector Machine (SVM) and Back Propagation Neural Network (BPNN) models had nonlinear fitting ability, but their generalization performance was limited under the small-sample and weak-feature scenario of this study, with test set accuracy lower than 77%. The ensemble learning model Extreme Gradient Boosting (XGBoost) achieved the highest test set accuracy of 77.78% among traditional models through ensemble fitting of multiple decision trees. Overall, none of the traditional machine learning models could simultaneously meet the industrial application requirements of high accuracy, fast speed, and strong generalizability for quail embryo sex identification.

Among deep learning models, the three convolutional architectures of 1D-CNN, 1D-ResNet, and CBAM-1DCNN achieved better discrimination accuracy than most traditional machine learning models via the local feature extraction capability of convolution kernels. The CBAM-1DCNN model integrated with the attention mechanism achieved the highest test set accuracy of 79.37% among all comparison models, but its single-sample discrimination time reached 3.215 ms (8.24 times that of our model), and it was prone to overfitting in small-sample scenarios, with insufficient generalization stability.

The GA-CARS-ELM model constructed in this study achieved 80.95% test set accuracy, with a difference of only 1.4 percentage points between validation set and test set accuracy, and a single-sample discrimination time of only 0.39 ms. Its comprehensive performance significantly outperformed all comparison models, with optimal recognition ability for weak sex-related Raman feature signals, excellent anti-overfitting performance, and adaptability to industrial high-throughput online detection.

### 3.5. Spectral Characteristic Analysis and Biological Significance of Sex-Related Characteristic Wavelengths

In this study, we performed Raman peak assignment on the screened core characteristic wavelengths, and analyzed the potential intrinsic correlation between characteristic signals and embryonic sex differences in combination with the molecular biological laws of quail embryonic sex differentiation, to elucidate the possible discrimination mechanism of the model at the molecular level. The Raman spectral signal of the inner shell membrane of quail embryonated eggs is essentially the superposition of vibration characteristics of biological molecules related to embryonic development and sex differentiation, with core signals concentrated in the characteristic vibration intervals of four categories of biomolecules: proteins, lipids, nucleic acids, and carbohydrates.

The 29 characteristic wavelengths screened by the CARS algorithm were mainly distributed in four characteristic intervals highly related to sex differentiation. The first was the amide characteristic band interval (1200–1350 cm^−1^ amide III band, 1600–1700 cm^−1^ amide I band), whose signals correspond to the characteristic vibration of protein peptide bonds and may be directly related to the expression levels of key sex differentiation regulatory enzymes including 3β-HSD and P450arom. The second was the lipid and steroid vibration interval (1000–1100 cm^−1^, 1720–1750 cm^−1^), corresponding to the skeleton vibration of phospholipids, cholesterol and steroid sex hormones. The third was the nucleic acid characteristic vibration interval (720–800 cm^−1^), reflecting metabolic differences in gonadal cell proliferation and differentiation between male and female embryos. The fourth was the carbohydrate vibration interval (900–950 cm^−1^), reflecting overall developmental and metabolic differences between male and female embryos.

**Figure 13:**
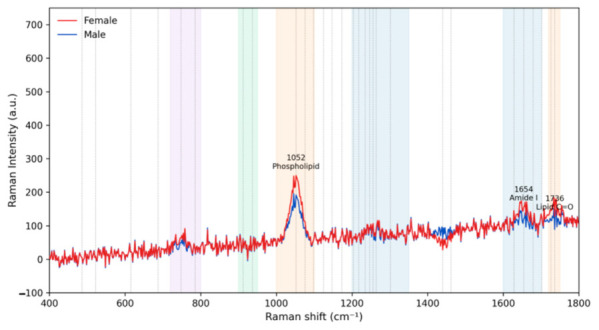
Comparison of average Raman spectra between female and male quail embryo samples and assignment of core characteristic peaks. (Red curve: average spectrum of female samples; blue curve: average spectrum of male samples; gray vertical dashed lines: positions of the 29 characteristic wavelengths screened by CARS; colored background blocks: four core sex-related characteristic intervals.).

We further compared the average spectra of valid male and female samples (Figure 13), and observed significant differences in signal intensity between male and female samples in the above core characteristic intervals. Statistical analysis using an independent samples *t*-test showed that the characteristic peak intensities of female embryos at 1654 cm^−1^ (amide I band, *p* < 0.01), 1736 cm^−1^ (lipid C=O stretching vibration, *p* < 0.01) and 1052 cm^−1^ (phospholipid C-O stretching vibration, *p* < 0.01) were significantly higher than those of male samples. This trend is consistent with the specific high expression law of key estrogen synthesis genes P450arom and 3β-HSD in female embryos on day 5 of incubation [20]. In contrast, male samples showed a higher signal response at the characteristic peak of 1440 cm^−1^ (lipid CH_2_ bending vibration related to androgen synthesis, *p* < 0.05). More than 70% of the characteristic wavelengths screened by the CARS algorithm were concentrated in the above four characteristic intervals with significant male–female differences.

These results confirmed that the characteristic wavelengths screened in this study effectively captured the molecular-level differences in sex differentiation of quail embryos, provided a plausible biological explanation for the high discrimination accuracy of the GA-CARS-ELM model, and also provided core targets for the subsequent identification of specific biomarkers for early sex differentiation of quail embryos.

## 4. Discussion

The global poultry industry faces an urgent demand for in ovo sexing technologies to resolve the resource waste and animal welfare crises caused by routine culling of day-old male chicks, yet existing research has long been limited to chicken embryos, leaving a critical technical gap for the quail farming sector.

Most established in ovo sexing studies, such as the Raman spectroscopic method for chicken embryos developed by Galli et al. [17], cannot be directly adapted to quail due to its 17-day short incubation cycle, which imposes far stricter requirements on early detection timing. Meanwhile, mainstream optical detection methods including near-infrared spectroscopy and hyperspectral imaging suffer from severe eggshell matrix signal interference [10,11,12,13,14,15], while PCR and hormone detection methods cause irreversible embryonic damage [8,9]. None of these existing technologies can balance detection accuracy, embryo protection, and industrial high-throughput application requirements.

This study makes three core practical contributions to fill these gaps. First, it establishes the first Raman spectroscopy-based in ovo sexing system for quail embryos, with a detection window on incubation day 5 that fully complies with avian embryo animal welfare principles. Second, the innovative “shell perforation without inner membrane damage” sampling strategy eliminates eggshell signal interference while minimizing embryonic damage, resolving the core contradiction between effective signal acquisition and embryo survival in industrial scenarios. Third, it elucidates the potential biological connotation of sex-specific spectral signals, providing key targets for the screening of avian sex differentiation biomarkers.

Nevertheless, the mechanical perforation method still has a certain impact on hatching rate, and the model’s cross-breed generalizability requires further validation. Additionally, the current manual operation limits the throughput of the system, and future automation of the perforation and spectral acquisition process will be essential for large-scale commercial application. Overall, this study provides a technically feasible and commercially applicable solution for pre-hatching sex sorting in quail farming, expanding the application boundary of Raman spectroscopy in poultry breeding research.

## 5. Conclusions and Outlook

This study establishes the first Raman spectroscopy-based analytical framework for non-destructive early sex identification of embryonated quail eggs, filling a long-standing critical research gap in in ovo sexing for small poultry species. The optimized GA-CARS-ELM model achieves a state-of-the-art comprehensive performance with a 80.95% test set accuracy and 0.39 ms single-sample detection speed, and we elucidate the plausible biological underpinnings of sex-specific spectral signals in quail embryos. This work provides a technically feasible solution for pre-hatching sex sorting in quail farming, directly addressing the industry’s core challenges of animal welfare controversy and resource waste.

Future research will focus on three core directions: optimizing sample preparation to improve embryo hatchability, identifying specific biomarkers for quail early sex differentiation, and developing automated industrial detection devices with multi-breed and multi-batch validation to advance technology translation.

## Figures and Tables

**Table 1:** Core parameters of Raman spectrum acquisition.

Parameter	Setting
Laser wavelength	785 nm
Spectral acquisition range	400–1800 cm^−1^
Laser aperture	50 μm
Grating scale	400 lines/mm
Spectral resolution	4.7–8.6 cm^−1^
Objective magnification	50×
Exposure time	10 s
Number of accumulative scans	3

**Table 2:** Sex distribution of valid samples in training, validation and test sets.

Dataset	Total Samples	Male Samples	Female Samples
Training set	188	95	93
Validation set	62	31	31
Test set	63	32	31
Total	313	158	155

**Table 3:** Comparison of modeling effects of different preprocessing methods and wavelength selection methods.

Preprocessing Method	No Preprocessing	SG Smoothing		MSC	SNV Transformation
Validation set accuracy of ELM model (%)	70.97	72.58		67.74	66.13
Wavelength selection method	-	CARS	SPA	-	-
Validation set accuracy of ELM model (%)	-	79.03	77.41	-	-

**Table 4:** Comprehensive performance comparison of different machine learning and deep learning models.

Model	Input Dataset	Test Set Accuracy (%)	Single-Sample Time (ms)	*p*-Value (vs. GA-CARS-ELM)
LR	Full wavelength	61.9	0.02	<0.001
	CARS	66.67	0.01	<0.001
	SPA	65.08	0.01	<0.001
SVM	Full wavelength	69.84	0.12	<0.001
	CARS	73.02	0.08	<0.001
	SPA	71.43	0.09	<0.001
BPNN	Full wavelength	71.43	0.25	<0.001
	CARS	74.6	0.18	<0.001
	SPA	71.43	0.19	<0.001
XGBoost	Full wavelength	74.6	0.32	<0.001
	CARS	77.78	0.27	0.021
	SPA	76.19	0.29	0.035
1D-CNN	Full wavelength	73.02	1.87	<0.001
	CARS	76.19	1.24	0.008
	SPA	74.6	1.31	0.012
1D-ResNet	Full wavelength	74.6	2.15	<0.001
	CARS	77.78	1.68	0.019
	SPA	76.19	1.75	0.028
CBAM-1DCNN	Full wavelength	76.19	3.52	<0.001
	CARS	79.37	3.21	0.047
	SPA	79.37	3.36	0.032
GA-ELM	Full wavelength	79.37	1.62	0.042
GA-CARS-ELM	CARS	80.95	0.39	-
GA-SPA-ELM	SPA	79.37	0.45	0.038

## Data Availability

The data presented in this study are available within the article. Further inquiries can be directed to the first author (Qian Yan, yqyq@webmail.hzau.edu.cn).
